# General practitioner hospitalists in psychiatry – may we help you?

**DOI:** 10.1192/j.eurpsy.2025.1841

**Published:** 2025-08-26

**Authors:** K. Mårtenson, M. Kojoukhova, E. Hallvar-Halonen, T. Talaslahti, M. Honkasalo, L. Rehnberg-Laiho, S. Eskelinen

**Affiliations:** 1 Psychiatry, University of Helsinki and Helsinki University Hospital, Helsinki, Finland

## Abstract

**Introduction:**

Patients admitted to psychiatric services present with several acute and long-term somatic health problems. Psychiatrists have limited time and expertise to manage those conditions. Nevertheless, general practitioner (GP) hospitalists rarely exist in psychiatric facilities.

**Objectives:**

To examine the effects of a novel hospitalist service we describe performance of GP hospitalists.

**Methods:**

HUS Helsinki University Hospital Psychiatry has 12 hospital campuses (550 beds in total) and over 30 outpatient clinics in Southern Finland. During February-May 2024 the organization had three part-time GP hospitalists covering 11 acute adult psychiatric wards (307 beds), six forensic psychiatric wards (120 beds), and two out-patient clinics. Hospitalist assessments at hospital wards and outpatient clinics included structured health checks and consultations from psychiatrists and registered nurses. The hospitalists collected characteristics from consecutive assessments by filling in an online survey. Somatic health conditions assessed in consultations, and those needing attention in health checks were coded according to ICD-10 classification.

**Results:**

The hospitalists provided 245 assessments: 223 consultations and 22 health checks. The majority (n=146,60%) of the assessments lasted for 30-90 minutes, one third (n=82,33%) lasted less than 30 minutes, while some (n=17,7%) took over 90 minutes. Of the assessments 49% (n=120) were hospitalist’s appointments, 12% (n=29) were provided by a phone call, 1% (n=3) were video appointments and 38% (n=93) were solved based on patient records. The most common conditions in the consultations were endocrine and cardiovascular related (Image 1). The hospitalists estimated that eight referrals to emergency departments and 22 to somatic specialists were avoided with the help of the consultations. In turn, hospitalists themselves referred 18 patients to somatic specialists. In the health checks the hospitalists identified 56 somatic conditions needing attention: cardiovascular, endocrine, gastrointestinal, dermatological and vision related problems were the most prevalent (Image 2).

**Image 1:**

**Figure:**
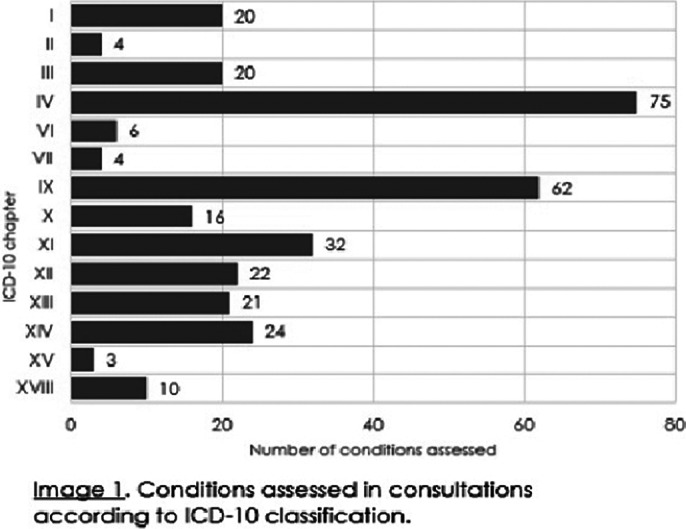

**Image 2:**

**Figure:**
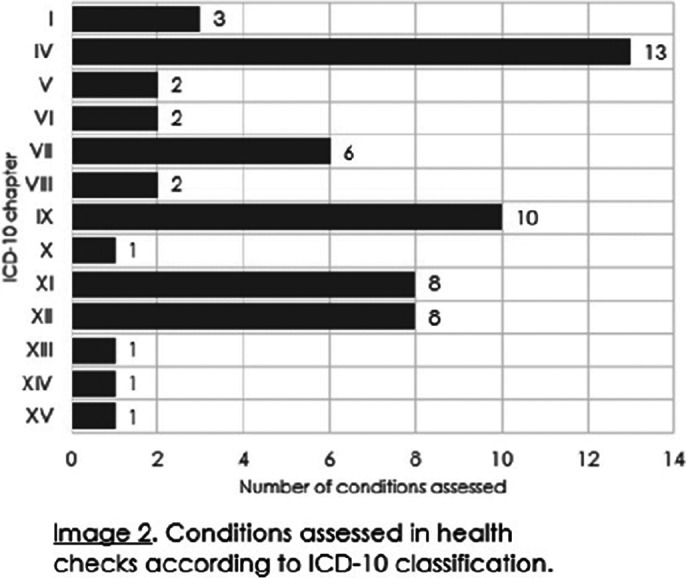

**Conclusions:**

The variety and amount of untreated chronic common diseases and specific somatic conditions related to psychiatric diseases/medications was substantial, and even for the GP hospitalists, time-consuming to handle. Cardiometabolic problems were the most prevalent of health concerns. GP hospitalists are one of the real-world solutions in improving the overall health of patients with severe mental illnesses, and in alleviating the heavy workload of treating psychiatrists.

**Disclosure of Interest:**

None Declared

